# Multi-functional properties of lactic acid bacteria strains derived from canine feces

**DOI:** 10.3389/fvets.2024.1404580

**Published:** 2024-08-05

**Authors:** Yunjiang Liu, Jiali Wang, Haohong Zheng, Jialiang Xin, Zhijun Zhong, Haifeng Liu, Hualin Fu, Ziyao Zhou, Xianmeng Qiu, Guangneng Peng

**Affiliations:** ^1^Key Laboratory of Animal Disease and Human Health of Sichuan Province, College of Veterinary Medicine, Sichuan Agricultural University, Chengdu, China; ^2^New Ruipeng Pet Healthcare Group Co., Ltd., Chengdu, China

**Keywords:** lactic acid bacteria, canines, probiotics, antimicrobial activity, adhesion, antioxidant capacity, metabolite

## Abstract

**Introduction:**

Probiotics, especially Lactic Acid Bacteria (LAB), can promote the health of host animals in a variety of ways, such as regulating intestinal flora and stimulating the host’s immune system.

**Methods:**

In this study, 206 LAB strains were isolated from 48 canine fecal samples. Eleven LAB strains were selected based on growth performance, acid and bile salt resistance. The 11 candidates underwent comprehensive evaluation for probiotic properties, including antipathogenic activity, adhesion, safety, antioxidant capacity, and metabolites.

**Results:**

The results of the antipathogenic activity tests showed that 11 LAB strains exhibited strong inhibitory effect and co-aggregation ability against four target pathogens (*E. coli*, *Staphylococcus aureus*, *Salmonella braenderup*, and *Pseudomonas aeruginosa*). The results of the adhesion test showed that the 11 LAB strains had high cell surface hydrophobicity, self-aggregation ability, biofilm-forming ability and adhesion ability to the Caco-2 cells. Among them, *Lactobacillus acidophilus* (L177) showed strong activity in various adhesion experiments. Safety tests showed that 11 LAB strains are sensitive to most antibiotics, with L102, L171, and L177 having the highest sensitivity rate at 85.71%, and no hemolysis occurred in all strains. Antioxidant test results showed that all strains showed good H_2_O_2_ tolerance, high scavenging capacity for 1, 1-diphenyl-2-trinitrophenylhydrazine (DPPH) and hydroxyl (OH^−^). In addition, 11 LAB strains can produce high levels of metabolites including exopolysaccharide (EPS), γ-aminobutyric acid (GABA), and bile salt hydrolase (BSH).

**Discussion:**

This study provides a thorough characterization of canine-derived LAB strains, highlighting their multifunctional potential as probiotics. The diverse capabilities of the strains make them promising candidates for canine dietary supplements, offering a holistic approach to canine health. Further research should validate their efficacy *in vivo* to ensure their practical application.

## Introduction

Dogs are the most commonly kept pets in households worldwide. However, they are susceptible to digestive system diseases, including gastroenteritis, pancreatitis, and inflammatory bowel disease due to changes in diet, environment, and weakened immune systems. These diseases can disrupt the body’s intestinal microbiota, leading to symptoms such as vomiting, diarrhea, allergies, and obesity (1). Antibiotics are often the preferred method for controlling these diseases (2, 3). However, excessive use of antibiotics can accelerate the development and spread of multi-drug-resistant bacteria, which poses a significant threat to disease treatment (4). A survey of drug resistance in intestinal bacteria isolated from domestic dogs revealed widespread antibiotic resistance, including resistance to enrofloxacin, penicillin, tetracycline, amoxicillin, oxacillin, erythromycin, and gentamicin (5–8). Additionally, extended use of antibiotics can weaken natural immunity and upset the balance of gut bacteria (9). As a result, alternative approaches are necessary to maintain the health of pet dogs.

Probiotics, specifically lactic acid bacteria (LAB), are often considered as potential alternatives to antibiotics due to their safety, high efficacy, little or no ability to develop drug resistance, and lack of toxic side effects. LAB are a group of bacteria that includes the genera Lactobacillus, Lactococcus, Pediococcus, Enterococcus, and Streptococcus, which are commonly found in feces and dairy-fermented foods (10). Numerous studies have demonstrated that LAB have various health benefits for the host, such as preventing infectious agents, regulating the immune system, reducing allergies and obesity, providing antioxidant capacity, increasing vitamin bioavailability, and reducing anxiety (1, 11–15). However, there is limited research on the probiotic function of LAB in domestic canines. Although studies have evaluated the tolerance of canine LAB to acids and bile salts, antibacterial activity, and antibiotic sensitivity (16–18), there is a lack of tests such as antioxidant and metabolite evaluation. Studies have shown that LAB can reduce the production of free radicals and damage to cells by maintaining the redox balance in the body (19). The evaluation of the antioxidant capacity of LAB in canines can enhance the clinical value of screening such bacteria.

While numerous studies have characterized probiotic strains for human use (20), there is a notable gap in the development and authorization of probiotic strains specifically for pets, such as dogs (21). Species specificity is crucial in probiotics, as strains isolated from the host species are more likely to colonize effectively and interact beneficially with the host’s microbiome (18). This study aims to address this gap by characterizing lactic acid bacteria (LAB) strains isolated from canine feces, with a focus on their potential as multifunctional probiotics tailored for canine use. Our research could contribute significantly to the development of effective and safe probiotics for dogs.

## Materials and methods

### Isolation purification and identification of LAB from canine feces

We collected fecal samples from 48 healthy adult dogs (1–5 years old) recruited from local veterinary clinics. All dogs were examined to ensure they were free from gastrointestinal diseases and had not received antibiotics or probiotics for at least 6 months. They were fed primarily commercial dry dog food and kept in home environments. Owners maintained the regular diet without additional supplements during the study. Fecal samples were collected in sterile containers, stored at 4°C, and processed within 24 h. Isolation and identification of LAB strains based on Zhang’s report (22). Each stool sample (1 g) was suspended in 10 mL of physiological saline after being crushed. Next, 0.1 mL of the suspension was applied to MRS (de Man, Rogosa and Sharpe Medium) agar plates (Hopebio, Qingdao, China) and incubated anaerobically at 37°C for 24 to 72 h until single colonies were obtained. The single colonies were purified through three consecutive passages on MRS agar. The single colonies were purified and enriched using MRS liquid medium. The bacterial cultures were then amplified and sequenced using 16S rDNA primers. Finally, the sequences were utilized for species identification through the BLAST function on the official NCBI website. The phylogenetic tree was constructed using MEGA11 software (Mega Limited, Auckland, New Zealand) for the sequences of isolated strains and homologous sequences, employing the Kimura 2-parameter model and the UPGMA method.

### Growth kinetics

Based on previous research (23), we tested the growth performance of LAB strains by constructing a growth curve using MRS broth as a negative control. Fifty microliters (1%) of each LAB strains culture in mid-exponential phase were inoculated into 50 mL of fresh MRS broth and incubated at 37°C for 48 h. The absorbance at 600 nm was measured every 2 h from 0 to 24 h and every 4 h from 25 to 48 h. From each type of LAB, we selected three strains with the best growth performance as candidate strains. After determining the growth period of the LAB strains, the OD600 value of the LAB was adjusted to approximately 0.1 before all subsequent experiments unless otherwise stated.

### Acid and bile salt tolerance

The Acid and Bile Salt Tolerance test was conducted based on Mayur with minor adjustments (24). The pH of the MRS broth medium was adjusted to 2.0, 3.0, and 4.0 using 0.1 mol/L HCl. The medium was then sterilized at 121°C for 20 min. The LAB strains were inoculated into the MRS medium and cultured until the late logarithmic phase. 1 mL of the culture was centrifuged and resuspended in an equal volume of MRS solution with different pH, and cultured in a 37°C incubator for 2 h. The plate count method was used to count the viable bacteria in the samples at 0 h (N0) and 2 h (N1). Samples were serially diluted, plated on MRS agar, and incubated anaerobically. Colony-forming units (CFUs) were then counted to determine the number of viable bacteria. Based on the counting results, the survival rate was calculated. Adjust the final concentrations of MRS broth medium to 0.1, 0.3, and 0.5% using bile salts (Solarbio, Beijing, China). The bile salt resistance test method is the same as the acid resistance test. Calculate the survival rate: survival rate (%) = N1/N0 × 100.

### Antipathogenic activity detection

#### Antibacterial activity

The Antipathogenic activity detection was conducted based on Zhang’s report with minor adjustments (22). The inhibitory capacity of cell-free supernatant (CFS) of LAB strains against four common enteropathogenic bacteria was determined using the Oxford cup method. The pathogenic bacteria for this test were *Staphylococcus aureus* ATCC 25923, *Escherichia coli* ATCC 25922, *Salmonella braenderup* H9812, and *Pseudomonas aeruginosa* PAO1. The LAB strain was activated and inoculated into MRS broth, cultured at 37°C for 24 h, and then centrifuged at 4500 rpm for 10 min under cooling. The supernatant was neutralized to pH 7.0 using sterile 1 M NaOH and filtered through a 0.22 μm sterile filter to obtain the CFS for later use. At the same time, 4 pathogenic bacteria were activated in LB broth under the same conditions. The pathogenic bacteria cultured to the stable phase were diluted to 10^7^ CFU/mL, and 100 μL was evenly spread on the LB solid medium for later use. Place 3 sterile Oxford cups on each culture medium, add 200 μL of CFS of the isolated strains into the Oxford cups respectively, use ordinary MRS liquid culture medium as a blank control, place the culture medium in a 37°C incubator for static culture 24 h, measure and record the diameter of the inhibition zone.

#### Co-aggregative ability with pathogens

Use the pathogenic strains mentioned in the Antibacterial activity test section to determine the co-aggregation ability of LAB strains. Mix the activated LAB strains Cultures with equal volumes of the four pathogenic bacterial cultures (2 mL each), vortex to mix, and incubate at 37°C for 2 h. The absorbance (*A*_mix_) of each mixed bacterial suspension was then measured at 600 nm. The absorbance of a single LAB strains suspension (*A*_LAB_) and pathogenic bacterial suspensions (*A*_pathogen_) was measured at 600 nm in the control group. For pathogenic bacteria, use the strains mentioned in the Antibacterial activity test section. The test was repeated three times and calculated according to the following formula: Co-aggregation rate (%) = 1 – *A*_mix_/[(*A*_LAB_ + *A*_pathogen_)/2] × 100.

### Adhesion activity detection

#### Auto-aggregation activity

The Auto-aggregation activity was conducted based on Zhang’s report with minor adjustments (22). The LAB strains cultured overnight was centrifuged at 4,500 r/min for 10 min to collect the cells. The cells were then washed twice with sterile 1 × PBS and adjusted to a concentration of 10^8^ CFU/mL before resting. The absorbance of the upper layer of the bacterial suspension was measured at 0 (*A*_0_) and 6 (*A*_1_) hours, respectively. The experiment was repeated three times. The rate of bacterial Auto-aggregation: Auto-aggregation rate (%) = 1 − (*A*_1_/*A*_0_) × 100.

#### Cell surface hydrophobicity

The activated LAB strains were introduced into MRS liquid medium at a concentration of 1% (v/v) and incubated overnight, collected the organisms by centrifugation at 8,000 × *g* for 10 min at 4°C and washed them three times with sterile 1 × PBS (pH = 7.4). The organisms were then resuspended in PBS and the absorbance of the LAB strains suspension was adjusted to OD600 = 0.60 ± 0.05 (A0). The hydrophobicity of LAB strains in various organic solvents was determined using the method reported by Kos et al. (25) with slight modifications. 1 mL of different organic solvents (Ethyl acetate, Xylol, and Trichloromethane) was added to 3 mL of LAB strains suspension, vortexed and shaken for 2 min, and then allowed to stand for 20 min, and then the OD value of the aqueous phase was measured at 600 nm by UV spectrophotometer (*A*_1_). The experiment was repeated three times. The hydrophobicity of LAB strains was calculated according to the following formula: hydrophobicity rate (%) = (1−*A*_1_/*A*_0_) × 100.

#### Adhesion to Caco-2 cells

The Adhesion to Caco-2 cells was conducted based on Wang’s report with minor adjustments (26). Colorectal adenocarcinoma cells (Caco-2) were purchased from CHINA CENTER FOR TYPE CULTURE COLLECTION, numbered GDC0153. Caco-2 cells were grown to a sub-confluent state of 80–90% in a cell culture flask, then digested with 0.25% trypsin, and counted using a hemocytometer. The concentration of viable cells was adjusted to 1 × 10^5^ cells/mL (*V*_C_) using DMEM medium. The cell suspension was added to a 12-well cell culture plate at a volume of 1 mL per well. The plate was then placed in a cell culture incubator at a constant temperature of 37°C and 5% CO_2_ for 48 h until the cells formed a monolayer. Cells were cultured for 1 day prior to the adhesion assay. Anti-resistant high-glucose DMEM was substituted for the medium at the beginning of the adhesion assay, the cells were washed three times with sterile PBS, and 1 mL of a LAB strains suspension at a concentration of 1 × 10^8^ CFU/mL (*V*_0_) was added to each well, 37°C and 5% CO_2_ in a constant-temperature cell culture incubator for 2 h. Following the incubation period, the cells underwent three washes with sterile PBS to eliminate any unattached LAB cells. Subsequently, 0.25% trypsin was used to digest the cells. Cells were collected and subjected to tenfold gradient dilution after complete digestion. Plate colony counting (*V*_1_) was used to determine the number of viable adherent LAB after dilution on MRS solid media. The experiment was conducted three times. The adhesion rate and adhesion index of LAB to Caco-2 cells were calculated using the following formula: Adhesion rate (%) = (*V*_1_/*V*_0_) × 100; Adhesion index (CFU/cell) = *V*_1_/*V*_C_.

### Determination of biofilm forming ability

Research shows that LAB strains with strong biofilm forming ability have better heat and freeze resistance (27). The ability of LAB strains to form a biofilm was determined by crystal violet staining (28). The LAB strains suspension in the lag phase was inoculated into a 96-well cell culture plate at 200 μL/well, and cultured in a 37°C incubator for 24 h to form a stable biofilm, and blank MRS liquid medium was used as a control. The bacteria were washed three times with sterile PBS to elute the planktonic bacteria, and then dried at room temperature for 15 min; fixed in methanol solution (200 μL) for 15 min and dried at room temperature for 10 min; stained in 1% crystal violet solution (200 μL) for 20 min, washed 3 times with distilled water, and dried at room temperature for 10 min; eluted in 33% acetic acid solution (200 μL) for 10 min. The OD value of the decolorized solution at 595 nm was measured by an enzyme counter (the control was recorded as *A*_0_ and the lactobacilli were recorded as *A*). The strength of biofilm formation ability of lactobacilli was evaluated according to the following criteria: no biofilm formation ability (−): *A*<*A*_0_; weak biofilm forming ability (+): *A*_0_<*A* ≤ 2*A*_0_; moderate biofilm forming ability (++): 2*A*_0_<*A* ≤ 4*A*_0_; and strong biofilm forming ability (+++): *A*>4*A*_0_. The experiments were repeated three times.

### Safety assessment

#### Hemolytic activity

The Safety assessment test was conducted based on Zhang’s report with minor adjustments (22). To assess hemolytic activity, LAB strains were streaked on blood agar plates and incubated for 48 h at 37°C. Staphylococcus aureus ATCC 25923 was used as a positive control. *β*-hemolysis, in which all erythrocytes are hydrolyzed, forming a clear area around the colony. *α*-hemolysis is when erythrocytes are partially hydrolyzed, forming a green area around the colony. *γ*-hemolysis occurs when erythrocytes are unresponsive and there is no hemolysis around the colony.

#### Antibiotic susceptibility

The antibiotic susceptibility of the selected LAB strains was assessed using the disk-diffusion test. Eighteen antimicrobials (Shunyoubio, Shanghai, China) were tested, including penicillin G (P, 10 μg), ampicillin (AMP, 10 μg), amoxicillin (AML, 25 μg), erythromycin (E, 15 μg), Cefuroxim (CXM, 30 μg), cefotaxime (CTX, 30 μg), Oxacillin (OX, 5 μg), Cefazolin (KZ, 30 μg), Norfloxacin (NOR, 5 μg), Rifampicin (RD, 5 μg), clindamycin (DA, 10 μg), chloramphenicol (C, 30 μg), tetracycline (TE, 30 μg), and vancomycin (VA, 30 μg). Fresh overnight cultures of each LAB strains were diluted to a concentration of 10^8^ CFU/mL. Subsequently, 100 μL of the diluted cultures were spread on MRS agar plates and dried. The LAB strains were tested for antibiotic susceptibility using the antibiotics listed above. Three uniform antibiotic disks were manually placed on the surface of the dried MRS plates, which were then inverted and incubated for 48 h under anaerobic conditions at 37°C. Antibiotic susceptibility was classified as resistant (R), moderately susceptible (M), or sensitive (S) based on the diameter of the zone of inhibition (mm) according to the parameters of the Clinical and Laboratory Standards Institute (29).

### Antioxidant capacity assessment

Inoculate activated LAB strains into the MRS liquid medium. After overnight culture, the mixture should be centrifuged at 4°C and 8,000 × *g* for 10 min. The supernatant should be collected to obtain a cell-free supernatant. The pellet should then be resuspended in PBS, and the concentration of cells should be adjusted to 1 × 10^9^ CFU/mL to obtain a bacterial suspension.

#### Tolerance to H_2_O_2_

The method reported by Xiong et al. (30) was used to measure the tolerance of LAB strains to H_2_O_2_. A liquid culture of LAB strains with a concentration of 1 × 10^8^ CFU/mL was inoculated into MRS liquid culture medium containing 0, 0.5, 1.0, 1.5, and 2.0 mmol/L H_2_O_2_ at an inoculum volume of 2% (v/v). The mixture was incubated for 8 h at 37°C in a constant temperature incubator, and the OD value of the culture medium was measured at a wavelength of 600 nm using a UV spectrophotometer. The experiment was repeated three times.

#### DPPH radical scavenging ability

The 1, 1-diphenyl-2-picrylhydrazyl (DPPH) free radical scavenging ability of LAB strains was detected according to the literature method (31). 2 mL of 0.2 mmol/L DPPH absolute ethanol solution was added to a centrifuge tube containing 1 mL of lactic acid bacteria cell-free supernatant or bacterial suspension. The mixture was vortexed and left to react for 30 min at room temperature in the dark at 4°C. After that, it was centrifuged at 8,000 × *g* for 10 min to collect the supernatant. The OD value of the supernatant was measured at a wavelength of 517 nm using a UV spectrophotometer (OD_sample_). Anhydrous ethanol was used as the blank group instead of DPPH absolute ethanol solution (OD_blank_), and distilled water was used as the control group instead of the sample for the reaction (OD_control_). The experiment was repeated three times, following that, the DPPH free radical scavenging rate of LAB was computed utilizing the subsequent formula: DPPH free radical scavenging rate (%) = [1−(OD_sample_ − OD_blank_)/OD_control_] × 100.

#### Determination of OH^−^ free radical scavenging ability

The determination of OH^−^ scavenging capability followed the protocol outlined by Alam et al. (32), with certain adjustments. A centrifuge tube received five hundred microliters of LAB strains cell-free supernatant or suspension. This was accompanied by the addition of 1 mL of 0.1% 1,10-phenanthroline, 1 mL of PBS, 1 mL of 2.5 mmol/L FeSO_4_, and 1 mL of 20 mmol/L H_2_O_2_. Following a 1.5-h incubation period in a water bath set at 37°C, the OD_536_ of the resultant reaction mixture was measured (OD_sample_). In the blank group, a consistent volume of absolute ethanol substituted H_2_O_2_ (OD_blank_). Similarly, in the control group, the sample solution was replaced with an equivalent volume of distilled water (OD_control_). The OH^−^ radical scavenging rate of LAB strains was determined by applying the following formula: OH^−^ free radical scavenging rate (%) = [(OD_sample_ − OD_control_)/(OD_blank_−OD_control_)] × 100.

#### Determination of O^2−^ free radical scavenging ability

The scavenging capacity of O^2−^ free radicals by LAB strains was assessed following the procedure detailed by Liu et al. (33). To 100 μL of LAB strains cell-free supernatant or bacterial suspension, 2.8 mL of 0.05 mol/L Tris–HCl (pH 8.2) and 100 μL of 0.05 mol/L pyrogallol were added. The mixture was vortexed and incubated at 25°C, shielded from light. After 4 min of incubation, the reaction was halted by the addition of 1 mL of 8 mol/L HCl. Use a UV spectrophotometer to measure the OD value of the reaction solution at a wavelength of 320 nm (OD_sample_). Adjust to zero with distilled water. Distilled water replaces the sample for reaction as a control group (OD_control_). The experiment was repeated three times. Then calculate the O^2−^ free radical scavenging rate of LAB strains according to the following formula: O^2−^ free radical scavenging rate (%) = [1−OD_sample_/OD_control_] × 100.

### Metabolite determination

#### Determination of exopolysaccharides (EPS) production capacity

The EPS production ability of LAB strains was determined according to the method reported by Ren et al. (34). LAB strains cell-free supernatant was prepared according to the method used in the antioxidant test. The supernatant was mixed with trichloroacetic acid to a final concentration of 40 mg/mL, incubated at 4°C overnight, and centrifuged to collect the upper aqueous phase (8,000 × *g*, 4°C, 10 min). Subsequently, 250 μL of 6% phenol and 1 mL of concentrated sulfuric acid were added to the collected liquid, mix well and incubate on ice for 1 min. The OD value of the reaction solution was measured at a wavelength of 490 nm using a microplate reader. A standard curve was drawn using glucose solutions with concentrations of 3.125, 6.25, 12.5, 25, 50, and 100 mg/L to calculate the concentration of EPS produced by LAB strains. The experiment was repeated three times.

#### Determination of gamma-aminobutyric acid (GABA) production ability

The GABA-producing ability of LAB strains was determined according to Zhang et al. (35). Firstly, the activated LAB strains were inoculated in glucose yeast extract peptone (GYP) medium, cultured overnight, and centrifuged at 4°C for 10 min at 8,000 × *g* to collect the supernatant. Subsequently, 200 μL of 0.2 mol/L borate buffer (pH 9.0), 1 mL of 6% phenol, and 0.4 mL of sodium hypochlorite solution with an available chlorine content of 5.5% were added to 0.5 mL of the supernatant. Finally, the supernatant containing the compound was boiled for 10 min, cooled in an ice bath for 20 min, and then mixed with 2 mL of a 60% ethanol solution by vortexing. The OD value of the reaction solution was measured at a wavelength of 645 nm using a microplate reader. A standard curve was drawn using GABA standards with concentrations of 0, 0.2, 0.4, 0.6, 0.8, and 1.0 g/L to calculate the concentration of GABA produced by LAB strains. The experiment was repeated three times.

#### Determination of bile salt hydrolase (BSH) producing ability

The ability of LAB strains to produce BSH was determined according to Wang et al. (36), and the cell-free supernatant was prepared according to the antioxidant test method. Firstly, 1 mL of bacterial suspension and 10 mmol/L dithiothreitol were mixed in a centrifuge tube and sonicated for 10 min. After that, the mixture was centrifuged at 4°C and 8,000 × *g* for 10 min to obtain cell-free extract. Next, 180 μL of PBS, 10 μL of 0.1 mol/L sodium taurocholate solution, and 10 μL of cell-free supernatant or cell-free extract were added in a centrifuge tube and heated in a 37°C water bath for 30 min. Then, 200 μL of 15% trichloroacetic acid was added. After reacting for 1 min, the mixture was centrifuged at 4°C and 8,000 × *g* for 10 min. Hundred microliter of supernatant was collected and mixed with 1.9 mL of ninhydrin chromogenic solution. Finally, after heating in a boiling water bath for 15 min and an ice water bath for 3 min, the OD570 of the reactant was measured to calculate the content of BSH produced by LAB strains by standard curve. In this test, trichloroacetic acid was first added to the sample, and then sodium taurocholate solution was added for reaction as a control group. A standard curve was constructed using glycine standards at concentrations of 0, 0.1, 0.2, 0.3, 0.4, and 0.5 μmol/L.

### Statistical analysis

All results were expressed as mean ± SD, and the statistical significance of the differences was evaluated by one-way ANOVA using SPSS 28 (IBM, United States), followed by Duncan’s multiple range test for *post hoc* analysis. Differences were considered significant at *p* < 0.05 and extremely significant at *p* < 0.01.

## Results

In this study, a total of 206 LAB strains were obtained from 48 canine fecal samples. Based on 16S rDNA identification, the bacterial species with the highest number of isolates was *Enterococcus faecalis* (122), followed by *Ligilactobacillus animalis* (16), *Limosilactobacillus reuteri* (14), *Enterococcus faecium* (14), *Weissella confusa* (14), *Ligilactobacillus salivarius* (6), *Weissella paramesenteroides* (5), *Lactobacillus acidophilus* (5), *Enterococcus hirae* (2), *Pediococcus acidilactici* (2), *Lactobacillus johnsonii* (2), *Limosilactobacillus fermentum* (1), *Ligilactobacillus agilis* (1), *Enterococcus lactis* (1), and *Lacticaseibacillus rhamnosus* (1).

**Figure 1:**
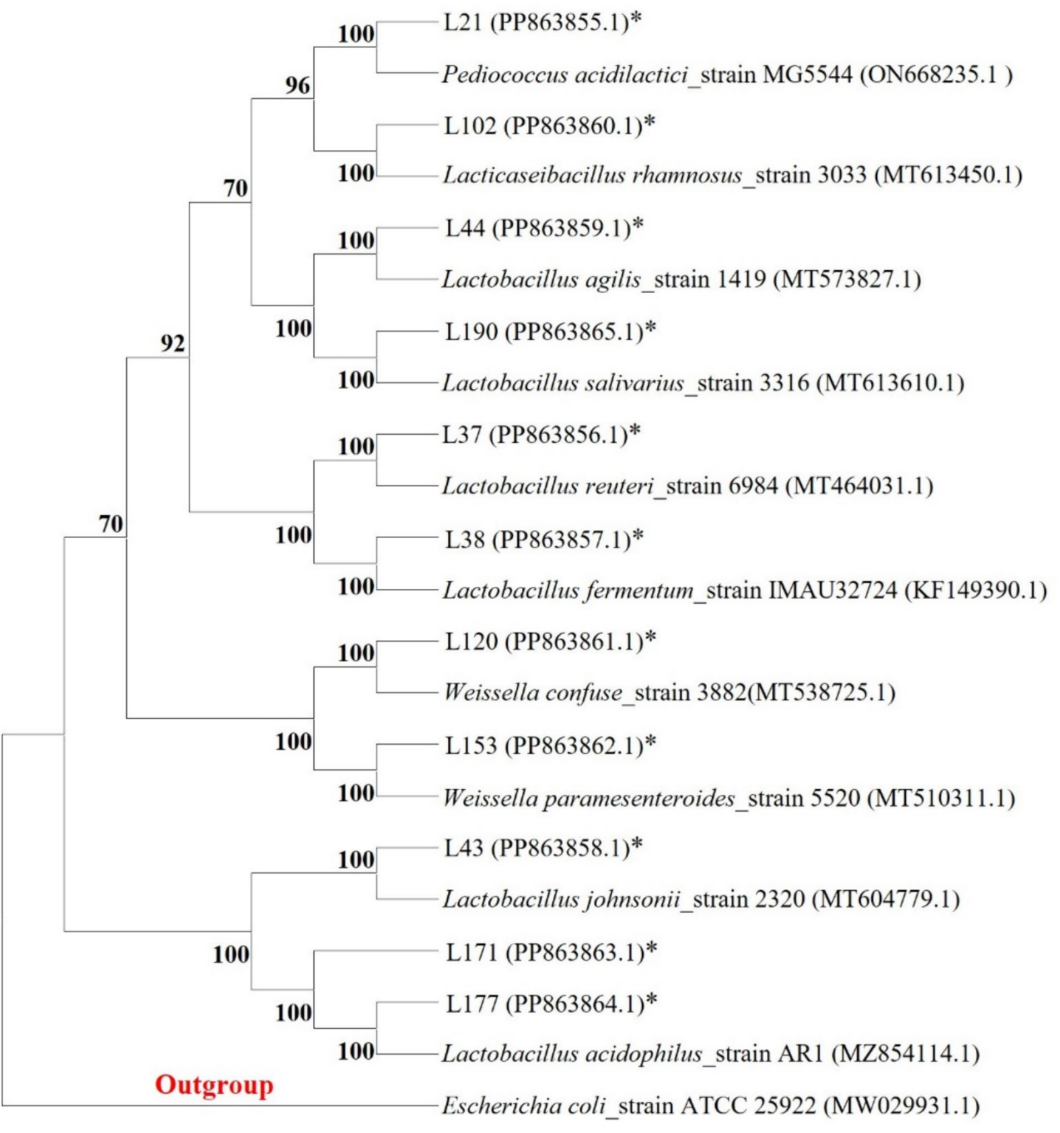
Phylogenetic tree of 11 LAB strains created with the data from 16S rRNA gene analysis results (Bootstrap value was 1,000 repeats; *Escherichia coli* ATCC 25922 was used as outgroup; *Represents the sequence of this study).

Among these strains, we selected 11 probiotic candidates based on their good growth performance and acid and bile salt resistance (growth performance data not shown). Table 1 presents information on 11 bacterial strains, including their species and corresponding numbers. Table 2 lists the survival rates of the 11 LAB strains at different pH and bile salt concentrations. The results showed that all strains had relatively good survival rates at pH = 4, and *Lactobacillus acidophilus* L77 had the highest survival rate of 86.34%. The bile salt resistance results showed that strains L43 and L153 had the highest survival rates at 0.1 and 0.3% bile salt concentrations with 58.15 and 23.67%, respectively. All strains showed no viability at 0.5% Bile salt concentration. The phylogenetic tree, constructed based on the 16S rDNA gene sequences and shown in Figure 1, provides a visual representation of the genetic relatedness among these isolates. These strains were evaluated for their probiotic properties, such as antipathogenic activity, adhesion, safety, antioxidant capacity, and metabolites.

**Table 1:** Numbering and species information of probiotic candidates.

Bacterial number	Species
L21	*Pediococcus acidilactici*
L37	*Limosilactobacillus reuteri*
L38	*Limosilactobacillus fermentum*
L43	*Lactobacillus johnsonii*
L44	*Ligilactobacillus agilis*
L102	*Lacticaseibacillus rhamnosus*
L120	*Weissella confusa*
L153	*Weissella paramesenteroides*
L171	*Lactobacillus acidophilus*
L177	*Lactobacillus acidophilus*
L190	*Ligilactobacillus salivarius*

**Table 2:** Survival rates of 11 LAB strains in different pH and bile salt concentrations.

Strain	Survival rate (%)				
	PH = 2	PH = 3	PH = 4	0.1% Bile salt	0.3% Bile salt
L21	7.26 ± 0.22^f^	15.09 ± 1.47^c^	73.88 ± 1.50^b^	10.51 ± 0.24^f^	21.42 ± 1.17^a^
L37	8.63 ± 0.48^cd^	9.94 ± 0.57^e^	21.93 ± 1.05^f^	23.89 ± 1.37^cd^	9.36 ± 0.36^d^
L38	8.11 ± 0.06^de^	60.94 ± 1.82^a^	71.57 ± 1.16^b^	15.46 ± 0.85^ef^	19.76 ± 1.63^ab^
L43	11.33 ± 0.66^b^	13.51 ± 1.32^cd^	25.98 ± 1.87^ef^	58.15 ± 1.01^a^	12.33 ± 0.85^cd^
L44	7.66 ± 0.63^ef^	8.10 ± 0.23^e^	40.46 ± 1.98^d^	20.98 ± 1.17^de^	7.86 ± 0.16^d^
L102	8.66 ± 0.11^cd^	9.48 ± 0.25^e^	27.32 ± 1.79^ef^	26.15 ± 1.48^cd^	16.31 ± 1.88^bc^
L120	13.49 ± 0.07^a^	15.53 ± 0.48^c^	34.38 ± 1.19^de^	44.41 ± 1.06^b^	15.43 ± 0.59^bc^
L153	8.16 ± 0.26d^e^	9.30 ± 0.16^e^	20.82 ± 1.14^f^	41.07 ± 0.53^b^	23.67 ± 1.05^a^
L171	7.72 ± 0.13^ef^	8.45 ± 0.30^e^	55.53 ± 1.34^c^	26.78 ± 1.71^cd^	8.18 ± 0.44^d^
L177	11.31 ± 0.34^b^	49.92 ± 1.50^b^	86.34 ± 1.95^a^	55.64 ± 1.42^a^	23.30 ± 0.50^a^
L190	9.17 ± 0.06^c^	11.07 ± 0.33^de^	63.61 ± 1.02^bc^	29.86 ± 1.00^c^	8.53 ± 0.26^d^

All the results are represented as mean ± SD. Different letters indicate significant differences (Waller-Duncan, *p* < 0.05) in the columns.

### Antipathogenic activity detection

To evaluate the antipathogen capabilities of these 11 candidates, we tested their antagonistic activity against common enteric pathogens, including *Escherichia coli* (ATCC 25922), *Staphylococcus aureus* (ATCC 25923), *Salmonella braenderup* (H9812), and *Pseudomonas aeruginosa* (PAO 1), using the Oxford cup method. Based on previous studies, the antipathogen activity of these LAB was classified into four ranges: I, 8 mm < area diameter ≤ 12 mm; II, 12 mm < area diameter ≤ 16 mm; III, 16 mm < area diameter ≤ 20 mm; IV, 20 mm < area diameter. As shown in Table 3, the inhibition zone diameters of the 11 strains were all greater than 10 mm, indicating that they all had significant antagonistic activity against four common intestinal pathogens, but their performances were not exactly the same. Among them, the bacteria with the strongest antibacterial effects against *Escherichia coli*, *Staphylococcus aureus*, *Salmonella braenderup*, and *Pseudomonas aeruginosa* are L38, L177, L177, and L43, respectively.

**Table 3:** Detection of antagonistic activity of LAB strains in canine fecal samples by the Oxford cup method.

Strain	Diameter of inhibition zone (mm)			
	*E. coli* ATCC 25922	*Staphylococcus aureus* ATCC 25923	*Salmonella braenderup* H9812	*P. aeruginosa* PAO 1
L21	14.41 ± 0.79^cde^	15.69 ± 0.58^ef^	13.44 ± 1.18^d^	20.32 ± 0.17^ef^
L37	14.76 ± 0.95^bcd^	14.60 ± 0.97^f^	17.85 ± 0.84^b^	23.99 ± 0.72^bc^
L38	17.49 ± 0.59^a^	17.27 ± 1.34^de^	10.99 ± 0.40^f^	24.73 ± 0.96^bc^
L43	16.57 ± 0.29^ab^	14.51 ± 1.41^f^	11.74 ± 0.62^def^	30.11 ± 0.55^a^
L44	12.71 ± 0.50^e^	18.91 ± 0.93^bc^	12.47 ± 1.32^def^	14.62 ± 0.42^g^
L102	12.85 ± 0.68^de^	20.17 ± 1.33^b^	12.96 ± 0.91^de^	12.53 ± 1.01^h^
L120	15.36 ± 1.03^bc^	16.45 ± 1.16^e^	11.33 ± 0.56^ef^	22.95 ± 0.71^cd^
L153	14.48 ± 1.26^cde^	16.86 ± 1.02^de^	11.96 ± 0.57^def^	21.67 ± 0.46^de^
L171	14.62 ± 0.93^bcde^	18.32 ± 0.98^cd^	16.61 ± 1.12^bc^	19.39 ± 0.92^f^
L177	15.90 ± 1.45^abc^	24.01 ± 0.87^a^	20.20 ± 0.67^a^	25.67 ± 0.60^b^
L190	15.58 ± 0.56^bc^	23.56 ± 0.23^a^	16.03 ± 0.88^c^	24.92 ± 0.56^bc^

All the results are represented as mean ± SD. Different letters indicate significant differences (Waller-Duncan, *p* < 0.05) in the columns.

Regarding co-aggregation activity, all 11 strains were able to co-aggregate the four pathogens as shown in Table 4. Among them, strain L177 has the strongest co-aggregation ability with *Escherichia coli* (ATCC 25922), *Salmonella braenderup* (H9812), and *P. aeruginosa* (PAO 1). It also has an extremely strong co-aggregation ability with *Staphylococcus aureus* (ATCC 25923).

**Table 4:** Co-aggregative activity of LAB strains from canine fecal samples against pathogenic bacteria.

Strain	Co-aggregative ratio (%)			
	*E. coli* ATCC 25922	*Staphylococcus aureus* ATCC 25923	*Salmonella braenderup* H9812	*P. aeruginosa* PAO 1
L21	67.75 ± 1.43^bc^	70.42 ± 1.43^bc^	69.05 ± 0.35^b^	69.64 ± 1.25^bc^
L37	66.98 ± 0.93^c^	68.94 ± 0.95^cd^	68.25 ± 0.51^b^	69.16 ± 0.66^bc^
L38	63.78 ± 1.00^de^	66.04 ± 1.72^e^	65.26 ± 1.34^c^	67.15 ± 1.04^c^
L43	66.44 ± 0.86^cd^	68.59 ± 0.96^cd^	67.75 ± 0.89^bc^	68.73 ± 1.02^c^
L44	67.19 ± 1.31^c^	69.67 ± 1.35^cd^	68.78 ± 0.86^b^	75.76 ± 0.99^a^
L102	49.00 ± 0.99^g^	58.16 ± 1.21^f^	49.07 ± 1.86^e^	52.90 ± 1.04^d^
L120	74.39 ± 1.04^a^	76.46 ± 0.78^a^	72.59 ± 0.83^a^	73.91 ± 0.87^ab^
L153	57.56 ± 1.51^f^	64.98 ± 1.21^e^	56.11 ± 0.83^d^	55.69 ± 1.01^d^
L171	63.57 ± 1.17^e^	65.89 ± 0.83^e^	67.34 ± 1.00^bc^	71.88 ± 1.29^abc^
L177	69.94 ± 0.43^b^	72.49 ± 0.47^b^	72.79 ± 1.68^a^	74.26 ± 0.15^ab^
L190	65.49 ± 1.34^cde^	67.33 ± 1.05^de^	66.25 ± 0.39^bc^	66.81 ± 0.72^c^

All the results are represented as mean ± SD. Different letters indicate significant differences (Waller-Duncan, *p* < 0.05) in the columns.

### Adhesion activity detection

Auto-aggregation Ability as shown in Table 5, strains L171 and L177 showed high auto-aggregation rates. Both exceeded 90%.

**Table 5:** Auto-aggregation abilities of 11 LAB strains isolated from canine fecal.

Strain	Auto-aggregation rate (%)
L21	27.84 ± 1.94^ef^
L37	70.95 ± 0.62^c^
L38	30.23 ± 3.6^e^
L43	29.77 ± 1.12^ef^
L44	72.36 ± 0.55^c^
L102	80.62 ± 0.87^b^
L120	43.1 ± 1.51^d^
L153	25.97 ± 0.92^f^
L171	92.35 ± 2.29^a^
L177	91.17 ± 1.41^a^
L190	40.28 ± 1.75^d^

All the results are represented as mean ± SD. Different letters indicate significant differences (Waller-Duncan, *p* < 0.05) in the columns.

The results of cell surface hydrophobicity are presented in Table 6. Significant differences were observed among the hydrophobicity rates of various LAB strains when exposed to different solutions. L177 exhibited the highest hydrophobicity rates to Ethyl acetate and Xylol. L171 exhibited the highest hydrophobicity rates to Trichloromethane.

**Table 6:** The cell surface hydrophobicity of 11 LAB strains isolated from canine fecal in different solutions.

Strain	The cell surface hydrophobicity rate (%)		
	Ethyl acetate	Xylol	Trichloromethane
L21	40.61 ± 0.33^c^	21.31 ± 0.85^f^	22.77 ± 2.81^g^
L37	49.64 ± 4.58^b^	37.07 ± 3.63^de^	87.27 ± 1.56^b^
L38	29.16 ± 0.31^de^	32.30 ± 2.41^ef^	35.02 ± 2.73^ef^
L43	35.92 ± 0.82^cd^	63.51 ± 3.94^bc^	72.33 ± 6.68^c^
L44	50.09 ± 1.68^b^	67.15 ± 2.54^b^	33.15 ± 5.89^f^
L102	53.65 ± 3.38^b^	22.96 ± 5.33^f^	87.06 ± 2.33^b^
L120	51.58 ± 2.90^b^	50.80 ± 2.74^cd^	21.59 ± 3.59^g^
L153	25.57 ± 3.36^e^	32.66 ± 4.05^ef^	47.60 ± 0.46^d^
L171	55.82 ± 0.22^b^	39.08 ± 5.22^de^	95.56 ± 0.19^a^
L177	83.27 ± 4.96^a^	80.79 ± 1.72^a^	82.76 ± 2.66^b^
L190	57.32 ± 1.05^b^	47.21 ± 5.23^d^	42.02 ± 0.83^de^

All the results are represented as mean ± SD. Different letters indicate significant differences (Waller-Duncan, p < 0.05) in the columns.

**Figure 2:**
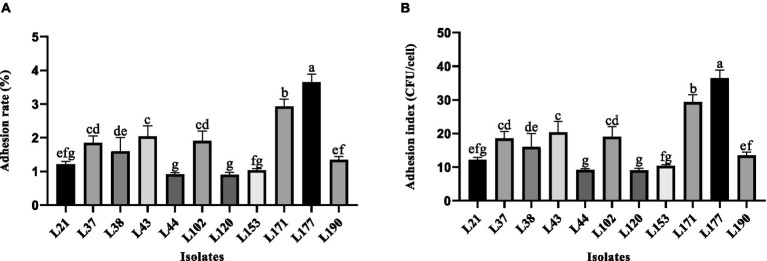
Adhesion ability of LAB strains to Caco-2 cell line. **(A)** Adhesion rate. **(B)** Adhesion index. Different letters indicate significant differences (Waller-Duncan, *p* < 0.05).

Figure 2 shows the variability in adhesion ability of the 11 LAB strains to the Caco-2 cells. Strain L177 exhibited the strongest adhesion ability (3.62% adhesion rate, 36.17 adhesion index), followed by strain L171 (2.82% adhesion rate, 28.17 adhesion index). It is noteworthy that both of these bacteria are *Lactobacillus acidophilus*.

Table 7 shows that all 11 LAB strains exhibited good biofilm-forming ability. Strains L21, L44, L153, and L171 exhibited moderate biofilm-forming ability (++), while strains L37, L38, L43, L102, L120, L177, and L190 exhibited strong biofilm-forming ability (+++).

**Table 7:** Biofilm formation capacity of 11 LAB strains isolated from canine fecal.

Strain	Biofilm formation capacity
L21	++
L37	+++
L38	+++
L43	+++
L44	++
L102	+++
L120	+++
L153	++
L171	++
L177	+++
L190	+++

### Safety assessment

To ensure *in vivo* applicability, all potential probiotics must be non-hemolytic. The hemolysis test results indicated that none of the 11 candidates were hemolytic, as they did not produce a β hemolytic loop in this experiment.

Table 8 shows the results of the evaluation of the susceptibility of 11 LAB strains to 14 commonly used antibiotics. The resistance rate (both resistant and intermediate) was 0% (0/11) for Penicillin G, Ampicillin, and Amoxicillin. For Erythromycin, the resistance rate was 27.27% (3/11), for Cefuroxim and Cefotaxime it was 9.09% (1/11), for Oxacillin it was 90.91% (10/11), for Cefazolin it was 0% (0/11), for Norfloxacin it was100% (11/11), for Rifampicin and Clindamycin it was 18.18% (2/11), for Chloramphenicol it was 0% (0/11), for Tetracycline it was 18.18% (2/11), for Vancomycin it was 72.73% (8/11). All isolates were more than 70% susceptible to 14 antibiotics. L102, L171, and L177 showed the highest susceptibility rate of 85.71%. The results of the inhibition zone diameters of the 11 LAB strains are shown in Supplementary Table S1.

**Table 8:** Antibiotic susceptibility of 11 LAB strains isolated from canine fecal to different antibiotics.

Strain	Antibiotic susceptibility														Sensitive rate (S + I, %)
	P	AMP	AML	E	CXM	CTX	OX	KZ	NOR	RD	DA	C	TE	VA	
L21	S	S	S	S	S	S	R	S	R	S	S	S	S	R	78.57
L37	S	S	S	S	S	S	R	S	R	S	S	S	S	R	78.57
L38	S	S	S	S	R	R	R	S	R	S	S	S	S	R	71.43
L43	S	S	S	R	S	S	S	S	R	S	R	S	I	S	78.57
L44	S	S	S	S	S	S	R	S	R	S	S	S	R	R	71.43
L102	S	S	S	S	S	S	R	S	I	S	S	S	S	R	85.71
L120	S	S	S	S	S	S	R	S	R	R	S	S	S	R	71.43
L153	S	S	S	S	S	S	R	S	R	I	R	S	S	R	71.43
L171	S	S	S	I	S	S	R	S	R	S	S	S	S	S	85.71
L177	S	S	S	I	S	S	R	S	R	S	S	S	S	S	85.71
L190	S	S	S	S	S	S	R	S	R	S	S	S	S	R	78.57

P, penicillin G; AMP, ampicillin, AML; amoxicillin; E, erythromycin; CXM, cefuroxim; CTX, cefotaxime; OX, oxacillin; KZ, cefazolin; NOR, norfloxacin; RD, rifampicin; DA, clindamycin; C, chloramphenicol; TE, tetracycline; VA, vancomycin.

### Antioxidant capacity assessment

#### Tolerance to H_2_O_2_

Tables 9, 10 show the antioxidant capacities of the 11 LAB strains, including tolerance to H_2_O_2_, DPPH radical scavenging capacity, hydroxyl radical scavenging capacity, superoxide anion scavenging capacity. Eleven LAB strains were found to survive in environments with varying concentrations of H_2_O_2_. However, their survival rate was relatively lower in 2 mmol/L H_2_O_2_ compared to lower concentrations. Among the isolates, L177 exhibited the strongest survival in environments with different H_2_O_2_ concentrations.

**Table 9:** Livability of potential probiotic strains from canine fecal samples in different concentrations of H_2_O_2_ environment.

Strain	Survival rate (%)			
	0.5 mmol/L H_2_O_2_	1.0 mmol/L H_2_O_2_	1.5 mmol/L H_2_O_2_	2.0 mmol/L H_2_O_2_
L21	101.54 ± 1.05^bc^	68.01 ± 0.68^c^	3.63 ± 0.04^d^	2.52 ± 0.03^c^
L37	99.85 ± 2.98^bc^	1.90 ± 0.06^i^	1.39 ± 0.07^f^	1.20 ± 0.10^e^
L38	100.31 ± 2.93^bc^	78.34 ± 2.07^a^	2.90 ± 0.09^e^	1.30 ± 0.07^e^
L43	86.84 ± 3.51^d^	7.03 ± 0.49^h^	2.92 ± 0.19^e^	1.96 ± 0.23^d^
L44	59.49 ± 3.30^e^	5.97 ± 0.33^h^	1.07 ± 0.04^fg^	0.76 ± 0.13^f^
L102	96.59 ± 3.09^c^	45.63 ± 1.79^e^	4.43 ± 0.88^c^	3.88 ± 0.26^b^
L120	104.30 ± 3.79^ab^	74.28 ± 2.46^b^	14.83 ± 0.42^b^	2.75 ± 0.08^c^
L153	86.34 ± 3.63^d^	24.49 ± 2.39^g^	0.49 ± 0.16^g^	0.28 ± 0.18^g^
L171	95.55 ± 3.43^c^	28.45 ± 3.95^f^	3.23 ± 0.01^de^	0.65 ± 0.02^f^
L177	110.44 ± 3.52^a^	80.83 ± 3.53^d^	19.26 ± 0.58^a^	5.27 ± 0.14^a^
L190	95.65 ± 3.23^c^	0.89 ± 0.06^i^	0.59 ± 0.17^g^	0.03 ± 0.02^h^

All the results are represented as mean ± SD. Different letters indicate significant differences (Waller-Duncan, *p* < 0.05) in the columns.

**Table 10:** DPPH, OH^−^, and O^2−^ radical scavenging activity of 11 LAB strains isolated from fecal samples.

Strain	DPPH scavenging rate (%)			OH^−^ scavenging rate (%)	
	Supernatant	Suspension		Supernatant	Suspension
L21	89.39 ± 1.43^a^	19.11 ± 1.29^g^		47.38 ± 1.65^e^	13.31 ± 0.76^g^
L37	86.87 ± 1.11^c^	25.94 ± 0.69^d^		61.87 ± 0.27^d^	33.07 ± 1.22^c^
L38	86.38 ± 1.9^c^	22.53 ± 0.13^e^		70.31 ± 1.34^bc^	24.25 ± 0.66^de^
L43	86.83 ± 1.11^c^	19.37 ± 0.22^g^		73.72 ± 2.16^abc^	22.36 ± 0.94^ef^
L44	86.00 ± 1.03^c^	67.06 ± 0.33^a^		67.41 ± 1.43^cd^	15.67 ± 0.64^g^
L102	87.32 ± 1.02^bc^	53.31 ± 0.14^c^		73.08 ± 2.58^abc^	14.12 ± 1.36^g^
L120	87.16 ± 0.78^bc^	20.16 ± 0.80^fg^		71.93 ± 2.72^abc^	61.44 ± 0.43^a^
L153	86.85 ± 0.80^c^	21.10 ± 0.40^f^		62.76 ± 2.17^d^	29.10 ± 0.98^cd^
L171	89.04 ± 0.79^ab^	20.26 ± 1.22^fg^		75.3 ± 1.34^ab^	41.00 ± 0.85^b^
L177	89.41 ± 0.93^a^	65.53 ± 0.76^b^		77.83 ± 3.49^a^	17.64 ± 1.45^fg^
L190	86.11 ± 1.04^c^	19.74 ± 0.64^g^		70.18 ± 1.63^bc^	27.80 ± 0.27^cde^

All the results are represented as mean ± SD. Different letters indicate significant differences (Waller-Duncan, *p* < 0.05) in the columns.

In free radical scavenging experiments, cell-free supernatants consistently outperformed bacterial suspensions. The DPPH scavenging rates of the bacterial suspensions ranged from 19.11 to 67.06%. The highest rate (67.06 ± 0.33) was observed in strain L44. The DPPH clearance of the supernatants generally remained in the range of 86.00 to 89.41%. The highest clearance (89.41 ± 0.93) was observed for strain L177. The OH^−^ clearance of the bacterial suspensions ranged from 13.31 to 61.44%, with strain L120 exhibiting the highest clearance (61.44 ± 0.43). The OH^−^ removal rates of the supernatants ranged from 47.38 to 77.83%, with strain L177 exhibiting the highest rate (77.834 ± 3.49). None of the isolated strains exhibited the ability to scavenge O^2−^, either in the bacterial suspension or in the cell-free supernatant.

### Metabolite determination

**Figure 3:**
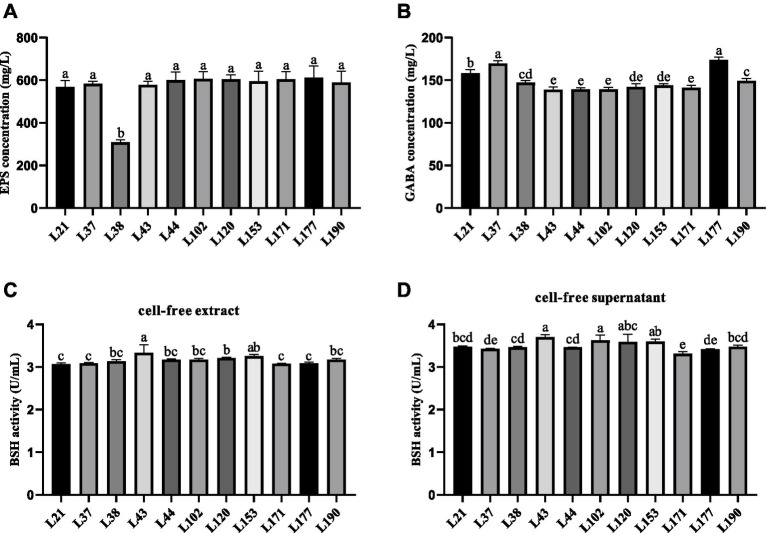
Metabolites production abilities of 11 LAB strains. All the results are represented as mean ± SD. **(A)** EPS production ability. **(B)** GABA production ability. **(C)** BSH production ability of cell-free extract. **(D)** BSH production ability of cell-free supernatant. Different letters indicate significant differences (Waller-Duncan, *p* < 0.05).

Figure 3 shows the results of metabolite evaluation for the 11 LAB strains. All strains, except for strain L38, exhibited an EPS production capacity of more than 550 mg/L, indicating good EPS production capacity (Figure 3A). The GABA-producing capacity of the isolates ranged from 139.09 to 173.79 mg/L, with strain L177 and L37 showed strong GABA production capabilities, which were 173.79 and 169.81 mg/L, respectively, (Figure 3B). The capacity of the cell-free supernatant to produce BSH was slightly better than that of the cell-free extracts, but there was not much variability among the strains. The BSH production capacity of the cell-free extract of the LAB strains ranged from 3.06 to 3.33 U/mL, Strain L43 showed the strongest BSH activity (Figure 2C). While the cell-free supernatant of the LAB strains ranged from 3.31 to 3.70 U/mL, Strain L43 also showed the strongest BSH activity (Figure 2D).

## Discussion

Currently, there is a growing interest in probiotics in veterinary medicine (37). Probiotics are an alternative to reduce the use of antibiotics and can treat and prevent infections, as well as gastrointestinal problems such as indigestion and vomiting in companion animals (38–40). However, the probiotics widely used in applied research in canines are mainly of non-canine origin, which can lead to poor efficacy due to homology issues (41). In this study, 11 candidates with good growth performance and acid and bile salt tolerance were screened from the feces of healthy dogs. This potential candidate properties were comprehensively characterized, including antipathogenic properties, adherence, safety, antioxidant activity, and metabolite profiling. The aim was to evaluate their possible use as canine probiotics.

Antipathogenic activity and safety properties are considered to be the most important properties for probiotic laboratories. In the present study, we tested the antagonistic activity of 11 candidates against 4 common enteric pathogens. Eleven LAB strains were found to inhibit the growth of all these pathogenic strains. Among them, the antibacterial activity of LAB against *Pseudomonas aeruginosa* strains is generally better than that against other pathogenic bacteria, which may be related to the production of some enzymes that can degrade *Pseudomonas aeruginosa* biofilms during the growth of LAB (42). In addition, *Pseudomonas aeruginosa* can cause various infections in dogs, including ulcerative keratitis, otitis, pyoderma, urinary tract infections, skin and respiratory tract infections (43). This suggests that our LAB candidates are expected to contribute to the treatment and prevention of these diseases in dogs in the future. Notably, in preliminary preparation we considered that aminoglycosides generally require aerobic metabolism for their optimal activity, so this antibiotic was not included in the antibiotic susceptibility testing portion of this study. However, although most LAB are anaerobic or facultative anaerobes, studies have shown that LAB exhibit considerable variability in their susceptibility to aminoglycosides (44, 45). Additionally, LAB can harbor aminoglycoside resistance genes (46). Therefore, in future final studies on LAB isolates, we will include aminoglycosides in the test group to fully evaluate the resistance of these isolates.

The LAB strains need to attach to the intestinal epithelial cells of the host to function in resisting invasion by pathogenic bacteria, maintaining balance within the intestinal microbiota, and modulating the immune response (47). This adhesion is correlated with cell surface hydrophobicity and Auto-aggregation activity. Enhanced cell surface hydrophobicity facilitates the interaction between LAB strains and epithelial cells, whereas auto-aggregation activity enables LAB strains to achieve high cell densities within the gut (48). In this study, 11 LAB strains derived from canines were found to be hydrophobic to organic solvents such as Ethyl acetate, Xylol, and Trichloromethane, suggesting that these strains may interact more closely with host epithelial cells, thereby enhancing their ability to positively influence host health. Additionally, these strains exhibited self-coagulation and adhesion to Caco-2 cells. Among the strains, *Lactobacillus acidophilus* (L177) showed the strongest characterization. Similar results were reported for *Lactobacillus acidophilus* M92 by Kos et al. (25). Probiotics in the periplasmic state have been shown to exhibit superior gastrointestinal tolerance and adherence capacity compared to probiotics in the free state (49). In this study, all 11 potential LAB strains exhibited robust biofilm-forming capabilities. These findings indicate that these strains possess the capacity to adhesion, colonize, and thrive within the gastrointestinal tract.

When the body undergoes oxidative stress, it produces large amounts of reactive oxygen species (ROS), including H_2_O_2_, DPPH, OH^−^, and O^2−^ radicals. Excess ROS attack proteins, lipids, nucleic acids, and other biomolecules, further exacerbating oxidative stress. Oxidative damage to these biomolecules can trigger apoptosis and is associated with a variety of diseases, such as inflammation, cancer, atherosclerosis, aging, and degenerative diseases (50). Multiple studies have demonstrated that LAB possess a potent antioxidant capacity, capable of inhibiting oxidative stress and mitigating the damage caused by associated diseases (51). LAB have been shown to exert their antioxidant capacity through both ROS scavenging and redox systems (19). It is important to note that the antioxidant capacity and mechanisms of different LAB species vary. In this study, the 11 canine-derived LAB strains demonstrated high tolerance to 0.5 and 1.0 mmol/L H_2_O_2_, as well as high scavenging capacity for DPPH and OH^−^ radicals. However, they did not show any O^2−^ radical scavenging capacity, which is consistent with the findings of Kuda et al. (52). Additionally, strains L120 and L177 demonstrated the ability to tolerate 1.5 mmol/L H_2_O_2_. Among the strains, L177 exhibited the strongest scavenging ability for DPPH and OH^−^ radicals. These results indicate that the 11 canine-derived LAB strains possess good antioxidant capacity, with *Lactobacillus acidophilus* L177 performing the best.

Beneficial metabolite production is a crucial factor in evaluating functional probiotics. EPS, produced by LAB during reproduction and metabolism, is an important metabolite that promotes animal health. Studies have shown that EPS can have beneficial effects on the organism through antibacterial, antiviral, antioxidant, antitumor, and immunomodulatory effects (53). Therefore, the screening for LAB strains that produce EPS and the quantitative analysis of EPS have garnered considerable attention. Hamet et al. (54) screened 28 strains of Lactobacillus spp. with EPS production capacities ranging from 20 to 370 mg/L. In this study, the 11 LAB strains demonstrated a strong EPS production capacity ranging from 308.39 to 612.78 mg/L, suggesting potential multifunctional effects. This study is one of the few to evaluate the EPS production capacity of LAB from canis. GABA is inhibitory neurotransmitter in the mammalian central nervous system. It has been investigated for its physiological roles, such as stimulating appetite, aiding digestion, managing epilepsy, suppressing cancer cell growth, and boosting immune function (55, 56). Therefore, the screening of GABA-producing LAB is a current research focus. However, there are few reports on GABA-producing LAB of canine origin. In this experiment, we discovered that 11 LAB strains produced 139.09–173.79 g/L of GABA, which is comparable to the 0.16 g/L produced by *Lactobacillus plantarum* 8,014 as reported by Li et al. (57). This suggests that the 11 LAB strains have the potential to be probiotics. Bile salt hydrolase (BSH) is an intracellular enzyme produced by intestinal flora during growth and reproduction. It regulates the balance of bile acids in the host, affects lipid metabolism, and controls cholesterol, as well as regulates intestinal diseases (58). Therefore, it is important to screen for LAB that produce BSH. Pinto et al. found that BSH activity was absent in all seven Lactobacillus isolates examined (59). Tsai et al. (60) screened 800 strains of Lactobacillus and found only 22 with BSH activity. In the present study, 11 LAB strains were found to have BSH activity, suggesting potential probiotic functions.

These findings suggest that the identified LAB strains, particularly *Lactobacillus acidophilus* (L177), hold promise as multifunctional probiotics for canine dietary supplements. Their diverse capabilities offer a holistic approach that may improve canine health by modulating intestinal flora, enhancing immune responses and providing antioxidant protection.

## Data availability statement

All strain sequencing data has been deposited in the NCBI database, accessible using the accession numbers: PP863855.1, ON668235.1, PP863860.1, MT613450.1, PP863859.1, MT573827.1, PP863865.1, MT613610.1, PP863856.1, MT464031.1, PP863857.1, KF149390.1, PP863861.1, MT538725.1, PP863862.1, MT510311.1 PP863858.1, MT604779.1, PP863863.1, PP863864.1, MZ854114.1, MW029931.1. Other data for this study are available upon reasonable request from the corresponding authors.

## Ethics statement

The animal studies were approved by Sichuan Agricultural University Animal Ethical and Welfare Committee. The studies were conducted in accordance with the local legislation and institutional requirements. Written informed consent was obtained from the owners for the participation of their animals in this study.

## Author contributions

YL: Conceptualization, Data curation, Formal analysis, Methodology, Software, Supervision, Writing – original draft, Writing – review & editing. JW: Investigation, Software, Writing – original draft. HZ: Data curation, Writing – original draft. JX: Methodology, Writing – original draft. ZhZ: Supervision, Writing – review & editing. HL: Supervision, Writing – review & editing. HF: Supervision, Writing – review & editing. ZiZ: Software, Supervision, Writing – review & editing. XQ: Supervision, Writing – review & editing. GP: Supervision, Writing – review & editing.
