# Supplementary material for: Multi-functional properties of lactic acid bacteria strains derived from canine feces
Source: Front Vet Sci. 2024 Aug 5;11:1404580. doi: 10.3389/fvets.2024.1404580 (PMC11330878; doi:10.3389/fvets.2024.1404580)
Supplement: Supplementary file 1 [file Table_1.DOCX]

Supplementary Material

# Supplementary Figures and Tables

## Supplementary Table

**Supplementary Table 1** Antibiotic susceptibility of 11 LAB strains isolated from canine fecal to different antibiotics

| Strain | Antibiotic susceptibility (mm) | | | | | | | | | | | | | |
| --- | --- | --- | --- | --- | --- | --- | --- | --- | --- | --- | --- | --- | --- | --- |
|  | P | AMP | AML | E | CXM | CTX | OX | KZ | NOR | RD | DA | C | TE | VA |
| L21 | 3.7 | 3.0 | 3.1 | 3.4 | 3.3 | 2.9 | 1.0 | 3.3 | 0.0 | 2.9 | 3.4 | 3.4 | 3.1 | 0.0 |
| L37 | 3.4 | 4.2 | 3.0 | 2.0 | 3.0 | 2.4 | 0.0 | 3.6 | 0.0 | 4.4 | 4.0 | 3.0 | 4.0 | 0.0 |
| L38 | 3.4 | 3.8 | 3.8 | 3.6 | 1.2 | 1.2 | 0.0 | 3.6 | 0.9 | 2.9 | 3.6 | 3.4 | 3.0 | 0.0 |
| L43 | 4.2 | 3.7 | 2.7 | 0.0 | 3.8 | 4.1 | 2.4 | 4.2 | 0.0 | 3.0 | 0.0 | 3.4 | 1.7 | 2.7 |
| L44 | 3.7 | 3.3 | 2.8 | 3.4 | 3.4 | 3.6 | 1.3 | 3.6 | 1.3 | 2.5 | 2.0 | 3.6 | 1.4 | 0.0 |
| L102 | 4.0 | 3.3 | 4.0 | 4.0 | 3.1 | 3.0 | 0.0 | 2.3 | 1.8 | 3.4 | 3.4 | 3.7 | 4.0 | 0.0 |
| L120 | 2.8 | 2.7 | 2.8 | 2.7 | 3.4 | 3.2 | 0.7 | 2.4 | 1.4 | 1.4 | 2.8 | 2.9 | 2.6 | 0.0 |
| L153 | 3.1 | 3.5 | 3.6 | 3.0 | 2.5 | 2.1 | 0.0 | 2.4 | 0.0 | 1.6 | 1.1 | 3.1 | 2.8 | 0.0 |
| L171 | 3.6 | 3.8 | 4.2 | 1.8 | 3.6 | 4.0 | 0.0 | 3.1 | 0.0 | 2.9 | 3.2 | 3.7 | 4.0 | 3.0 |
| L177 | 3.8 | 3.4 | 4.2 | 1.5 | 3.4 | 3.9 | 0.0 | 2.9 | 0.0 | 2.8 | 2.5 | 3.4 | 3.6 | 3.0 |
| L190 | 4.0 | 4.1 | 3.2 | 3.4 | 4.3 | 3.5 | 1.0 | 4.0 | 1.3 | 4.3 | 3.2 | 3.6 | 3.2 | 0.0 |

P, penicillin G; AMP, ampicillin, AML; amoxicillin; E, erythromycin; CXM, Cefuroxim; CTX, cefotaxime; OX, Oxacillin; KZ, Cefazolin; NOR, Norfloxacin; RD, Rifampicin; DA, clindamycin; C, chloramphenicol; TE, tetracycline; VA, vancomycin.
